# Enhancing investigative interview training using a child avatar system: a comparative study of interactive environments

**DOI:** 10.1038/s41598-023-47368-2

**Published:** 2023-11-21

**Authors:** Syed Zohaib Hassan, Saeed Shafiee Sabet, Michael Alexander Riegler, Gunn Astrid Baugerud, Hayley Ko, Pegah Salehi, Ragnhild Klingenberg Røed, Miriam Johnson, Pål Halvorsen

**Affiliations:** 1 Department of Holistic Systems, SimulaMet, 0167 Oslo, Norway; 2 Faculty of Technology, Art and Design, OsloMet, 0167 Oslo, Norway; 3 Faculty of Social Sciences, OsloMet, 0167 Oslo, Norway; 4 Faculty of Health Sciences, OsloMet, 0167 Oslo, Norway; 5 Oral Health Centre of Expertise in Eastern Norway, 0369 Oslo, Norway

**Keywords:** Computer science, Information technology

## Abstract

The impact of investigative interviews by police and Child Protective Services (CPS) on abused children can be profound, making effective training vital. Quality in these interviews often falls short and current training programs are insufficient in enabling adherence to best practice. We present a system for simulating an interactive environment with alleged abuse victims using a child avatar. The purpose of the system is to improve the quality of investigative interviewing by providing a realistic and engaging training experience for police and CPS personnel. We conducted a user study to assess the efficacy of four interactive platforms: VR, 2D desktop, audio, and text chat. CPS workers and child welfare students rated the quality of experience (QoE), realism, responsiveness, immersion, and flow. We also evaluated perceived learning impact, engagement in learning, self-efficacy, and alignment with best practice guidelines. Our findings indicate VR as superior in four out of five quality aspects, with 66% participants favoring it for immersive, realistic training. Quality of questions posed is crucial to these interviews. Distinguishing between appropriate and inappropriate questions, we achieved 87% balanced accuracy in providing effective feedback using our question classification model. Furthermore, CPS professionals demonstrated superior interview quality compared to non-professionals, independent of the platform.

## Introduction

Child abuse is a global concern that is known to have adverse effects on children’s development and mental and physical health. Research has shown that children who are victims of abuse experience cognitive impairments, and both mental and physical health issues that can affect them throughout their entire lives^1^. In addition, meta-analytic studies have estimated that 22.6$\%$ of children experience physical abuse and 11.8% are subjected to sexual abuse before the age of eighteen^2,3^, i.e., 8% of boys and 19% of girls face child sexual abuse (CSA)^4^. However, Andrews et al.^5^ argue that CSA prevalence actually ranges from 2-62$\%$ for sexually abused children during childhood, with the reported prevalence varying due to different methods of data collection and assessing CSA, meaning that the incidence of CSA may be higher.

Less than 15% of reported CSA cases have physical evidence, with only 9% of cases having medical evidence^6^. Furthermore, studies have shown that in 70% of the CSA cases children are the only witnesses of the criminal incident^7,8^. Thus, investigative interviews of children are critical for the prosecution of these cases. Research has further shown that children are reliable witnesses when interviewed properly and in line with best-practice recommendations^9–12^, i.e., developed by researchers and professionals to establish rapport and increase narrative details with the child^13^. These guidelines promote communication with the child through open-ended questions (e.g., “Tell me what happened.”), active listening, and support^14,15^. To encourage children to express themselves in their own words, encourage elaborate and coherent information, and prevent any contamination of their original memory, interviewers should use open-ended questions^16^. However, multiple international studies have found that these guidelines are rarely followed and that the quality of investigative interviews is quite poor^17–20^. The need to enhance the quality of investigative interviewing is evident and widely acknowledged in academic discourse. While some training programs have shown promise, they have not been effective in consistently improving interviewer behavior^21–23^. Recent studies, including one conducted in Norway over a 10-year period, suggest that despite advancements in training and innovation, there has been no significant improvement in interview quality^24^. To address this issue, it may be useful to design an interactive training program that can effectively train professionals in investigative interviewing.

This is precisely where the domain of computer science, specifically artificial intelligence (AI), can offer invaluable contributions. To the best of our knowledge, there are currently two child avatar training systems with the primary objective of enhancing the quality of interviews. Linnæus University and AvBIT Labs in Sweden developed an interview training system that employs prerecorded audio and video responses of an abused child. A human operator selects an appropriate video response for the user, which is displayed through Wirecast software controls on the Skype interface^25,26^.

Pomedda et al.^27^ presented Empowering Interviewer Training (EIT), an investigative interview training platform that uses a rule-based algorithm to select a pre-defined response based on user input. The user is presented with pre-recorded videos of children displaying different emotions, selected by a human operator. They conducted multiple studies with EIT system and investigated the impact of feedback on training effectiveness using their system and conducted multiple studies to analyze its learning effects^28–31^. Their findings suggest that incorporating feedback enhances learning effects, but the system has limited response generation capabilities.

While these systems have contributed to enhancing investigative interviewing abilities, they still possess certain limitations. Their response generation is inflexible or requires human intervention in their operation, leading to greater operational costs and the possibility of human errors. We have developed a training system for investigative interviews that incorporates lifelike avatars capable of dynamically responding to various queries, providing an immersive experience through virtual reality (VR) technology^32^. Our approach utilizes advanced natural language processing (NLP), which enables computers to understand and generate human language, and vision technologies, which allow machines to interpret and generate visual content, in combination to achieve this goal to create a virtual talking avatar. These technologies synergize to create a virtual talking avatar that simulates an abused child^33^.

The utility of VR extends beyond mere immersion. Increasingly, research indicates that VR can be a powerful tool for learning and education. Compared to 2D, audio, chatbot, or other interactive environments, VR can create a more engaging environment. Pausch et al.^34^ along with Bailenson et al^35^. discuss the use of immersive VR in educational settings and its impact on engagement and motivation. Both sets of authors concur that the immersive qualities of VR can elevate engagement and motivation, potentially leading to increased practice time. However, Pausch et al.^34^ also caution that over-immersion can sidetrack users from learning goals, diminishing the effectiveness of learning. Similarly, Bailenson et al.^35^ stress the significance of aligning VR experiences with learning objectives and balancing immersion and learning outcomes to optimize educational benefits. Numerous studies have been conducted to assess the impact of VR on learning in comparison to alternative methods. Lai et al.^36^ examined a vocabulary learning game in both VR and 2D, concluding that VR resulted in a significantly higher mean Quality of Experience (QoE) than the 2D desktop for vocabulary learning. Krokos et al.^37^ obtained similar outcomes for a memory learning task. In contrast, Madden et al.^38^ conducted a comparative study on learning moon phases through VR, PC, and hands-on training. They reported no significant difference between the three environments.

Studies have also explored the effectiveness of VR interview training platforms in improving interview skills and vocational outcomes, primarily focusing on individuals with conditions like autism spectrum disorder (ASD), schizophrenia, and substance use disorders. These studies consistently reveal positive results, including enhanced interview skills, boosted self-confidence, and a higher rate of job offers for participants who underwent VR interview training^39–41^. However, it is important to note that the efficacy of VR interview training may vary depending on the specific population and context in which it is used. Using VR for investigative interviews with artificial children is a complex issue that requires investigation. Adaptability and acceptability of VR are also key issues, which are influenced by various social groups based on gender or profession. Raaen et al.^42^ examine the level of acceptance and utilization of VR technology among the Norwegian population. The study reveals that merely 20% of the population has had any prior experience with VR, and only 0.6% of the individuals use it once per week. The feasibility of using VR for investigative interviews with artificial children is a complex issue that requires further investigation, especially in the context of the QoE for CPS workers.

In our earlier work^32^, a pilot QoE study was conducted with child protective services (CPS) professionals, who are experts in interviewing abused children, where they interacted with a virtual child avatar in VR. QoE measures the degree of delight or annoyance of the user of an application or service^43^. The results showed that the perceived realism, encompassing aspects such as appearance, speech, and lip-sync, along with a sense of presence, feeling fully immersed and engaged in an environment^44,45^, were crucial factors for the users. To further investigate these aspects of realism, another user study was conducted^46,47^. Realism study’s findings reveal the presence of an uncanny valley, wherein participants favored interacting with animated avatars over highly realistic avatars generated by GAN technology^48^. Additionally, the research demonstrated that employing natural voices for virtual avatars did not enhance realism or improve user experiences when compared to computer-generated voices. Participants in the pilot study also expressed the importance of note-taking during long-duration interviews, and it remains a challenge to develop a VR environment where users can take notes.

Furthermore, previous research did not focus on domain experts, and as demonstrated by Sabet et al.^49^, domain experts have different perceptions and expectations of a child avatar system than non-experts. Domain experts have specialized knowledge, training, and experience in dealing with real-world situations involving children, which may shape their expectations and responses when interacting with virtual child avatars in a sensitive context. For instance, experts may have a more nuanced understanding of the non-verbal cues and behavioral patterns of children, which can influence their assessment of the realism and effectiveness of virtual child avatars for interviews. It is essential to conduct further research to explore the feasibility and potential benefits and risks of using VR for investigative interviews with artificial children. This motivated our comparative study to assess the QoE of expert participants in various interactive environments. The aim of our research is to investigate the possibility and potential advantages and drawbacks of utilizing VR as a means of conducting investigative interviews with artificial children, in comparison to other interactive mediums like 2D, audio, and text. Our study involved participation from CPS workers and child welfare psychology students, referred to as non-CPS in our paper.

The first part of the study design involves participants engaging with a child avatar for 90 seconds in various environments and sharing their feedback on the experience. ITU-T, which stands for the International Telecommunication Union’s Telecommunication Standardization Sector, provides recommendations through ITU-T P.809 for conducting subjective experiments aimed at assessing the QoE for gaming services^50^. According to recommendations, short interactive sessions are suitable for measuring overall QoE, but may not be sufficient for assessing flow and immersion for sessions lasting less than 10 to 12 minutes. We argue, however, that it could be adequate for comparing different stimuli within the same setup. In the second part of the study, we also ask participants to interact with the child avatar just in VR environment without any time constraints. The motivation for second part came from the positive feedback we received from the pilot study^32^, where participants gave high ratings for quality metrics and QoE in the VR environment. Our aim was to gather more data on the VR experience in investigative interview training and investigate any disparities in short and long interactive sessions in VR. Furthermore, we sought to confirm our hypothesis that brief interactive sessions are sufficient for comparing stimuli in different environments. Both groups, CPS and non-CPS, took part in both the first and second parts of the study, and their data were collectively analyzed.

The system architecture uses the dialogue model described in^32^ with a new persona of a child. In addition to VR in current system architecture, for this study we also added three additional interactive environments (2D desktop, audio and text). We focused on identifying the optimal interactive environment to enhance the learning outcomes of the investigative interview training system. We do not evaluate the dialogue model or the participants’ ability to conduct investigative interviews during the user study. The findings of this study have implications for the training of police and CPS personnel in investigative interviewing. We aim to provide a more engaging and realistic training experience, which can lead to better learning outcomes and higher motivation for practice.

In investigative interview training, it is crucial to provide trainees with feedback that can help them improve their questioning techniques. Accurately distinguishing between recommended and not recommended questions, the training program can provide trainees with targeted feedback, enabling them to learn from their mistakes and improve their questioning techniques^51^. So apart from conducting the user study, we introduce and validate our classification model that can assess the quality of interviews by categorizing the questions as either open-ended or closed-ended. This model will be integrated into the system and provide feedback to the participants on how did they perform. In this paper, we focus on validating the performance of our classification model and conducting a comparative analysis of the interview quality between two groups: CPS and non-CPS. To the best our knowledge, Haginoya et al.’s model^52^ is the only relevant work with which we can compare our classification model’s performance. They present an automated question classification system for simulated CSA interviews using avatar. The system was found to work well in classifying interviewer questions, and automated interventions (feedback and modeling) were provided to improve interview quality. They managed to achieve an accuracy of 70% for their classifier. In their study, the intervention groups showed improvement in their second interview while the no intervention group did not. In their research, the authors utilize N-gram features in combination with an XGBoost classifier. N-gram refers to a grouping of N consecutive characters, words, or phrases within a given text. For instance, if we apply bigrams to the question “tell me everything” we extract three distinct combinations: “tell me,” “me everything,” and “everything about,” each of which represents a pair of adjacent words. In this case, we assign a frequency value of “1” to each of these bigrams to indicate their occurrence within the text and use it as features for training machine learning model. XGBoost, an abbreviation for Extreme Gradient Boosting, is a powerful machine learning algorithm employed in both classification and regression tasks. It operates by aggregating the predictions of numerous weak classifiers to make decisions.) Their methodology is constrained by its limited contextual window around a given word. This approach, however, falls short in its ability to comprehensively understand and contextualize entire sentences.

While addressing the limitation in previous work our paper aimed to address following questions:

- How do different environments influence the users’ QoE, presence, flow, realism, responsiveness, and learning experience?
- Does different environments affect the quality of the interview?
- Are 90 seconds enough for the comparison of different stimuli and to capture user experience for different system modalities?

In this paper, we build upon our work earlier work^53^ where we provided preliminary findings for the first part of the comparative study that we conducted. Specifically, we delve deeper into the qualitative feedback received from the study participants to gain a better understanding of their experiences. Furthermore, we introduce a feedback classification model and analyze its performance to better understand its potential applications. The main contributions of our work presented in this paper are:

- The development of a new dialogue model with a persona resembling a child that was exposed to sexual abuse.
- Extending the prototype of the child avatar in VR to three more environments of 2D, audio, and text.
- Conducting a comparative study for the four different interactive environments and evaluating them based on multiple quality experiences which captures user experience.
- A binary classification model, that can learn to recognize patterns in the interview questions and categorize them as either closed-ended or open-ended to assess the quality of interviews

## Results

In this section, we present the results of part 1 of our study in which we compared the user experience in four different interactive environments, namely VR, 2D, audio, and text, over a 90-second period. We analyzed the participants’ subjective feedback about each environment, as well as the quality of questions asked in each environment. Furthermore, as part two of our study, we evaluated the participants’ experience in the VR environment during an unrestricted session. Additionally, we assessed a classification model’s ability to distinguish between open-ended and close-ended questions.

### Study Part-1

**Figure 1:**
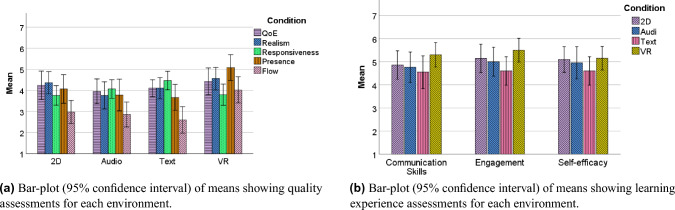
User experience.

We use five quality assessment metrics namely, QoE, realism, responsiveness, presence and flow to compare the user experience in each environment. We employ the Mean Opinion Score (MOS) to gauge the comprehensive quality assessment of a system. MOS is derived by averaging the individual ratings provided by participants for each quality metric. Figure 1 shows that VR has an higher average ratings for each quality metrics except responsiveness. We discussed the detailed results for each metric in this section.

#### Quality of experience

QoE is the degree of delight or annoyance of the user of an application or service, which results from the fulfillment of the users’ expectations with respect to the utility and/or enjoyment of the service ^43^. Figure 1a shows participants’ overall QoE ratings were averaged across conditions. We observe that VR created a slightly higher overall QoE ($M = 4.50, SD = 1.40$), followed by 2D ($M = 4.25, SD = 1.52$), Text ($M = 4.10, SD = 0.85$) and Audio ($M = 3.95, SD = 1.32$). However, a one-way repeated measured Analysis of variance (ANOVA), with a significance level of $\alpha = 0.05$, finds no significant differences ($F (3.0,17.0) = 1.115, p = 0.371$) between the MOS of overall QoE across different environments.

#### Presence

The quality of presence refers to a psychological state where an individual feels fully immersed and engaged in an environment, as if they are physically present in that space^44,45^. To evaluate any significant effects of the environments on the sense of presence, a one-way repeated measure ANOVA is used. The result of $F (3.0,18.0) = 5.528, p = 0.007$ indicates a significant effect. The post-hoc tests followed by a Bonferroni correction indicate that, as we expected, VR ($M = 5.08$, $SD = 1.35$) creates a significantly higher presence than 2D ($M = 4.06$, $SD = 1.49$, $p = 0.048$) and Audio ($M = 3.78$, $SD = 1.64$, $p = 0.005$), but surprisingly not compared to Text ($M = 3.87$, $SD = 1.59$, $p = 0.070$). No other pairwise comparisons were found to be significant.

#### Realism

The realism score is the mean of three questions regarding realism in the *appearance*, *talking*, and *lip-sync* of the avatar, mentioned in methods section. Distinct questionnaires were used to assess the realism of VR and 2D in comparison to audio and text, primarily due to the visual element involved. The impact of different environments on the perceived realism of the avatar was investigated using a one-way repeated measure ANOVA. The result $F (3.0,17.0) = 3.532, p = 0.037$ indicates a significant difference. The Text environment had a MOS of $(M) = 4.10$, $(SD) = 1.07$, Audio had a $M = 3.75$, $SD = 1.45$, 2D had a $M = 4.35$, $SD = 1.18$ and VR scored the highest ($M = 4.57$, $SD = 1.20$). The post-hoc test followed by a Bonferroni correction indicates that there is a significant difference for the VR-Audio pair ($p = 0.033$), but not for any other pairs.

#### Flow

Flow refers to a state of optimal experience in which a player is fully immersed and engaged in an interactive environment. It is a key indicator of a positive experience, as it indicates that the user is fully engaged and enjoying the experience^54^. For the flow, one-way repeated measure ANOVA indicates a significant impact of environments ($F (3.0,17.0) = 9.020, p = 0.001$). Text had a MOS of $(M) = 2.60$, $(SD) = 1.33$, Audio had a $M = 2.78$, $SD = 1.28$, 2D had a $M = 2.88$, $SD = 1.13$ and VR the highest score of $M = 4.00$, $SD = 1.40$. The post-hoc tests followed by a Bonferroni correction indicate that VR was rated significantly higher than 2D ($p = 0.03$), Audio ($p = 0.001$), and Text ($p = 0.008$), but there were no significant differences between any other pairs.

#### Responsiveness

Responsiveness describes the temporal aspects of the feedback a player receives after performing an action^55^. The quicker the response time, the more responsive the environment is regarded. Moreover, the fluidity of the visual and audio feedback also contributes to the responsiveness, impacting the overall experience^56^. One-way repeated measure ANOVA of responsiveness for the avatar in each environment does not show a significant difference among them ($F (3.0,17.0) = 2.893, p = 0.064$). The highest MOS was achieved by text ($(M) = 4.63$, $(SD) = 1.19$), followed by Audio ($M = 4.06$, $SD = 0.96$), VR ($M = 3.79$, $SD = 1.10$) and 2D ($M = 3.76$, $SD = 1.01$). This can be attributed to the STT and TTS synthesis in the environment with the audio element involved.

#### Learning experience

Apart from questions relating to user experience, we also incorporated three questions that pertained to the learning experience, which can be found in methods section. Figure 1b displays the average rating for these three questions. To investigate whether diverse environments could result in varying learning experiences, a one-way repeated measures ANOVA was conducted. Table 1 reveals that the different environments did not have any significant impact on communication and self-efficacy. Nevertheless, there was a noteworthy disparity between the perceived user engagement during learning across the various environments. A pairwise post-hoc comparison followed by a Bonferroni correction demonstrated that VR led to significantly higher engagement compared to Audio ($p = 0.022$) and Text ($p = 0.020$), but not when compared to 2D ($p = 0.85$). No significant differences were detected between any other pairs.

**Table 1:** The results of one-way ANOVA tests comparing the means of learning experience aspects in different environments. The mean difference is significant at the .05 level.

Metric	*VR*	2*D*	*F*-value	*p*-value
Communication skills	3	2.211	2.174	0.113
Engagement	3	1.99	4.422	0.015*
Self efficacy	3	2.311	2.174	0.179

#### Sensitivity analysis and effect size comparisons

We further scrutinized our user study’s data through sensitivity analysis to gauge the robustness of our findings. The sensitivity analysis underscored that, with our sample size of 21 and a desired power of 0.8, the smallest effect size (*f*) our study could reliably detect when comparing four different environments was approximately 0.81. Table 2 shows the observed effects for each metric, derived from the ANOVA test.

Comparing these observed effect sizes to our desired effect highlighted potential limitations of our study. The observed effect size for *QoE* and *self-efficay* was considerably below the desired effect. Metrics such as *presence*, *responsiveness*, and *engagement* displayed effect sizes that, while meaningful, were below the threshold of what our study was optimally powered to detect. In contrast, *flow* demonstrated stronger effects, suggesting that our sample size was more aligned with capturing such effects. An observed effect size that is less than the desired effect size does not invalidate your findings. It suggests that there is a higher risk of Type II errors which imply failing to detect an effect that is there. Conducting a future study with a larger sample size can reduce the risk of errors.

**Table 2:** Partial eta squared $(\eta ^2_p)$ and cohen’s *f* score calculated using sum of squares of effects ($SS_{effect}$) and sum of squares of error ($SS_{error}$) from ANOVA test.

Metric/source	$SS_{effect}$	$SS_{error}$	$\eta ^2_p$	Cohen’s *f*
QoE	3.3	55.7	0.056	0.059
Presence	22.619	93.881	0.194	0.241
Realism	7.383	39.283	0.158	0.188
Flow	24.213	52.038	0.318	0.465
Responsiveness	10.296	43.093	0.193	0.239
Comm.skills	6.789	6.211	0.108	0.121
Engagement	10.338	44.413	0.189	0.233
Self-efficacy	4.5	49.5	0.0833	0.0909

### Study Part-2

**Figure 2:**
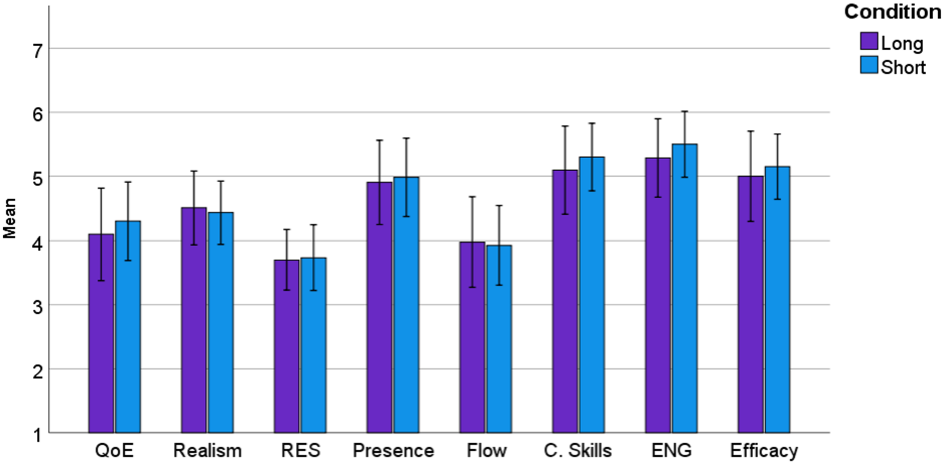
Bar-plot (95% confidence interval) of means showing quality assessments for VR session of 90-second VR termed short and without any time constrained termed as long. RES, C.Skills and ENG refers to responsiveness, communication skills and engagement respectivel.

The motivation behind this study was to assess the overall user experience of participants in the absence of time constraints. Additionally, the study aimed to validate our design decision that a 90-second duration is sufficient for users to engage with an interactive environment effectively and provide reliable feedback. Figure 2 shows that there is no significant difference in participants’ ratings of the quality aspects between the 90-second VR session and the unconstrained session in VR. Table 3 shows the results of the repeated measure t-test between the long and short VR experience across different quality metrics, which included QoE, realism, responsiveness, presence, and flow.There were no significant differences observed in the assessment metrics, when comparing short stimulus tests to longer sessions for VR. This suggests that a 90-second short stimulus is sufficient for measuring these quality metrics for each interactive environment.

**Table 3:** The results of one-way ANOVA tests comparing the means of learning experience aspects in different environments. The mean difference is significant at the .05 level.

Metric	Environment	Paired Samples Statistics		Paired Samples Test	
		Mean	Std. Deviation	t	Sig. (2-tailed)
Overall Exp	Long	4.0952	1.57812	− 1.046	0.308
	Short	4.4286	1.39898		
Realism	Long	4.5079	1.25883	− 0.349	0.731
	Short	4.5556	1.16587		
Responsiveness	Long	3.6984	1.03228	− 0.573	0.573
	Short	3.7937	1.09786		
Presence	Long	4.9048	1.44585	− 0.757	0.458
	Short	5.0794	1.34951		
Flow	Long	3.9762	1.54496	− 0.197	0.846
	Short	4.0238	1.36452		
Comm.skills	Long	5.0000	1.48678	− 1.552	0.137
	Short	5.3000	1.12858		
Eng	Long	5.2857	1.34695	− 1.552	0.055
	Short	5.5714	1.12122		
Self-efficacy	Long	5.0000	1.54919	− 1.096	0.286
	Short	5.2381	1.13599		

### Classification model

The assessment was conducted on 40 mock transcripts, containing 400 closed and 2,015 open-ended questions. Table 4a presents the performance evaluation of three classification models on test data. The models are random baseline classifier which generates predictions on assumption that classes are uniformly distributed, the model by Haginoya et al.^52^, and our model. The metrics used to evaluate the models include macro F1-score, weighted F1-score, balanced accuracy, and Matthews correlation coefficient (MCC).

From Table 4a, we can observe that the dummy model, which randomly predicts labels, has the lowest performance in all metrics. The Haginoya et al.’s model has better performance than the dummy model, but it is not clear from the reported data how it performs relative to the our proposed model since they only reported the accuracy score which we assume is not balanced accuracy and rest of the metrics are missing. Our model still outperforms the Haginoya’s models in basic accuracy metric.

The performance of the proposed classification model was further evaluated for each class. Table 4b presents a per-class analysis of our model’s performance on test data. The classes are closed and open questions, and the metrics used to evaluate the performance are precision, recall, and F1-score.

**Figure 3:**
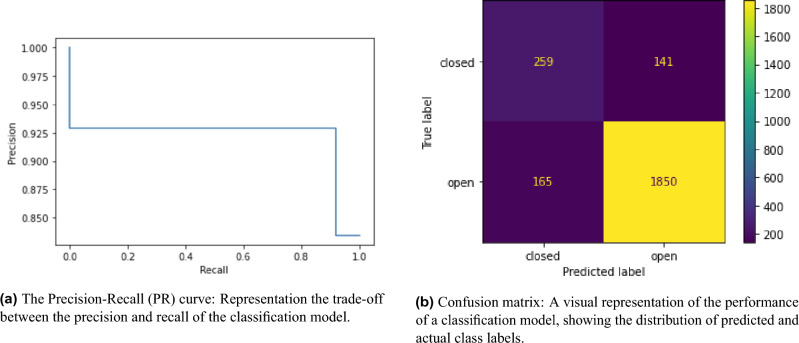
Qualitative analysis of classification model performance.

From table 4b, we can observe that the proposed model performs better for the open class than the closed class, as indicated by higher precision, recall, and F1-score values for the open class. The model’s precision for the closed class is lower than that for the open class, which means that there are more false positive predictions for the closed class. The model’s recall for the closed class is lower than that for the open class, which means that there are more false negative predictions for the closed class. Figure 3b illustrates that false negatives (FN) are more frequent for closed-ended questions than open-ended ones.

Overall, the proposed model’s performance is good, but there is room for improvement in predicting the closed class. We are convinced that transforming it into a multi-label task will enhance generalization and reduce false predictions. According to Lamb et al.^51^, the inter-coder reliability rate among experts for coding investigative interviews is approximately 85%. While our classification model outperforms other automated coding methodologies, as stated in Haginoya et al.’s research^52^, our goal is to achieve expert-level precision.

**Table 4:** Evaluation of the classification model: Test Data Classification Report.

(a) Our model performs better than the Haginoya’s binary classifier in distinguishing between open-ended and close-ended questions			
Metrics	Baseline	Haginoya^52^	Our model
Macro F1-score	0.44	?	**0.78**
Weighted F1-score	0.57	?	**0.87**
Balanced accuracy	0.48	0.72 (Normal)	**0.87**
MCC	− 0.02	?	**0.55**

(b) Per-Class analysis of the model performance: Evaluating on test data			
Metrics	Closed	Open	
Precision	0.61	0.93	
Recall	0.65	0.92	
F1-score	0.63	0.92	

Best results are highlighted using bold fonts. “?” represents that information is not available.

The model’s performance, as indicated by the metrics we used is quite promising, with a significant improvement over the Dummy model and potentially better performance than the Haginoya model. Despite the stepped appearance of the PRC, which suggests some limitations in the model’s ability to distinguish between classes or a lack of diverse confidence score distributions, the overall metrics demonstrate good performance. The model has high precision, recall, and F1-scores for both classes, indicating a good balance between correctly identifying true positives and minimizing false positives. Additionally, balanced accuracy and MCC values further support the ability of the model in classifying both positive and negative classes. While the Precision-Recall curve’s (PRC), in figure 3a, appearance suggests potential areas for improvement, the model’s performance is quite effective based on the provided metrics and the fact that the dataset is biased.

### Quality of interviews

**Figure 4:**
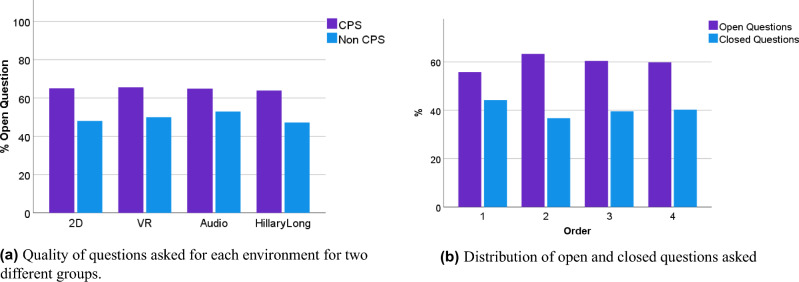
Quality of Interviews.

Table 5 presents the average number of questions asked in each environment. The average number of turns in the *text* environment was limited to 4.5 and Long VR was 29.7, due to being an outlier they are removed from the following data analyses, which focus on comparing the quality of the interviewers’ questions between different environments. Figure 4a shows the percentage of open-ended questions asked between CPS and non-CPS participants in different environments. Overall, 64% of the questions asked by CPS participants were open-ended, while for non-CPS it was 48%. That shows, as expected, experts conduct higher-quality interviews. However, the number of open-ended questions asked could have been higher among CPS.

Furthermore, no difference between the environment and quality of the interview even when split by CPS versus non CPS, which shows the higher cognitive load created in environments with visual elements does not distract the users from conducting interviews. Figure 4b shows no significant impact of the order in which participants interact with environments over the quality of the interviews.

A chi-square test of independence was performed to evaluate the relationship between the training environment (2D, audio, VR short and VR long) and question type (open versus closed-ended questions). The relationship between these variables was not significant, $X^2(3)=0.143, p = 0.986$. Thus, the training environment did not affect the quality of the interviews.

**Table 5:** The average number of questions asked in the 90s interview for each environment.

Environment	Number of turns
Text	4.5
2D	7.82
VR	8.17
Audio	8.52
Long VR	29.70

### Qualitative results

A 4-item post-test questionnaire was utilised, as shown in Table 9, which was then followed by an open question asking participants to provide their reasons behind their selection. A top-down coding approach was utilised to analyse the post-test questionnaire, as shown in Table 6. Many of the participants selected VR as their preferred environment. 76% of the participants perceived VR as the most realistic environment, 66% preferred VR over other environments, and 61% agreed that VR created the best experience for them. Participants with previous VR experience showed a preference for VR – 80% rated VR favourable in the post-test questionnaire. However, also 75% of the participants with no VR experience preferred VR over the other environments, and 60% rated VR as the environment that they liked, preferred, and created the best experience.

In addition, a thematic analysis was conducted to examine the responses of the open question regarding the participants’ experiences related to the different training environments. The following themes, *Sentiment towards environment* and *Realism* were explored.

*Sentiment towards environment* addressed the participants’ feelings about using the training program in 2D, audio, text, and VR environments. Sentiments were categorized into positive or negative feelings and were examined by whether the participant worked within CPS or not. While discussing participants’ feedback, we add extra text in ’[ ]’ to their responses, to adjust the tense and make participants’ feedback grammatically correct. CPS participants only expressed negative sentiments regarding the audio environment. Participants noted that audio “felt strange as [they] mostly talk to children face-to-face”, that “it [was] difficult to read body language” and that it “miss[es] the non-verbal communication”. Regarding the text environment, CPS participants were generally positive, stating that it felt familiar and that it “can be a good way to practice, as there will be many times when you also work on chat”. However, a non-CPS participant felt like “it [was] difficult to interact with the avatar in text”. A few participants expressed that they preferred the 2D environment as it has both the visual and body language of the child, and they are used to working with PCs. CPS participants had positive experiences with 2D, with a participant expressing that “2D felt more familiar” and it was “even better than audio, because it’s better to see the child [they were] talking to”. The VR environment generated the most positive responses, with many participants expressing that the realism of the avatar made them feel like it was easier to talk to the child. However, one CPS participant stated that this environment “[did] not really reflect [their] experience with having these kinds of conversations...because it seem[ed] a bit scripted...like you have to have the right kind of question to get a reply”.

Participants also reflected on the *Realism* of the interactive training program. Most participants stated that VR created the most realistic environment compared to the other environments. One participant stated that VR “made [them] feel like [they were] in the same room as Hillary” and that the realism made it “easier to talk to the child”. However, one participant noted that despite the realism, they quote “didn’t connect with Hillary”. These views were similar in regards to the 2D environment, with participants agreeing that it felt realistic but not as realistic as VR. Regarding the audio and text environments, participants stated that these “environments did not feel as real” compared to the other environments. A few participants expressed that although the text is not as realistic as VR, it can be a better environment for training as they are used to this way of communication compared to VR. One participant noted that although “text only was not very realistic, [it] can work when training”. Overall, the participants’ descriptions gave the impression that realism is an important aspect of an interactive training program.

Overall, the participants responded quite positively towards their experiences in this study. One participant “believe[ed] this way to practice is a good option [for] training in the school setting”. However, some participants had difficulties using the program as one participant stated that “it would have been much easier if everything was in Norwegian” and that “it took some time to formulate and write in English”. Only one participant reported a negative overall experience, stating that it was “frustrating all together, the system did not give me any responses that [were] useful in VR, audio, and 2D”.

**Table 6:** The results of the user study to evaluate the number of votes in response to the questions.

Items	VR	2D	Audio	Text
Q1-Which environment felt more realistic for you?	16	2	1	2
Q2-Which environment do you like the most?	14	3	1	3
Q3-Which environment do you prefer to use?	14	3	1	3
Q4-Which environment creates the best experience for you?	13	3	2	3

## Discussion

Our study aimed to compare the quality and learning aspects of experts in four different environments. Interactions lasted for 90 seconds in each environment. We observed that audio-based environments had more conversational turns than the text environments, as participants typed slower than they spoke. Despite a consistent dialogue model used in all environments, participants’ experiences were influenced by the responses they received from the model. Environments with fewer responses limited participants’ information, potentially affecting the richness and depth of interactions. As a consequence, the results of the study could have been impacted by this. Responses from the avatar were shaped by the questions asked, and because the questions themselves could vary, they also had the potential to influence participants’ experiences across different environments. It is worth noting that although the conversation’s quality may differ, we did not evaluate the dialogue model in this study.

Our findings indicate that VR was rated higher in four out of five quality assessments used for comparative analysis. However, there was no significant difference in the overall rated QoE for each environment. As anticipated, participants rated VR highest in terms of presence, indicating a greater sense of being part of the environment compared to the other options. VR also generated a significantly greater flow for users, creating an immersive environment that caused participants to lose track of time and forget their surroundings. These quality aspects may enhance users’ willingness to engage, potentially leading to longer practice sessions with the child avatar system. When examining the quantitative analysis for realism, the only noteworthy variation was found between the VR-audio pair. The text environment was rated highest in responsiveness due to the lack of delay because of TTS and STT synthesis, and participants were in control of when to send their messages. In addition, as shown in Figure 1b, all three metrics measuring the learning experience have a relatively higher mean for VR. Of particular significance is the higher user engagement for VR compared to audio and text environments. Mulqueeny et al.^57^ highlight the importance of engagement in achieving long-term learning and outcomes. With significantly greater engagement and the highest average values across all learning experience metrics, VR may lead to better learning and knowledge transfer. The majority of participants preferred VR as their interactive environment for the interview training system, which was likely influenced by the fact that most users were using VR for the first time and found it fascinating.

Nevertheless, it is premature to assert that VR is the superior environment at this stage. While VR offers better levels of realism, presence, and flow, about 40% of the participants preferred other environments due to familiarity, accessibility, and regularity of use. Moreover, it is important to note the size of our study with the two different groups of our participants, CPS students and CPS professionals. We found both of these groups to be well-suited for participation in our study due to their shared knowledge, training, and experience in conducting interviews with children. Recruiting participants with relevant experience was a crucial and difficult job. When we looked closely at our results using sensitivity analysis, we found something crucial. While our findings are significant and provide valuable insights into the various metrics, the study might not have been optimally powered to detect certain nuanced effects, especially for specific metrics. This is why we aim to continue our research with more participants to get a clearer picture. Additionally, future studies should look more closely at each group. This could help us to see if different levels of expertise lead to different results. As we continue our research, these insights will be essential to help us improve our future studies even better.

The quality of the questions plays a crucial role in determining the effectiveness of an interview. Open-ended questions tend to elicit more informative responses, while closed-ended questions can yield potentially inaccurate answers. A system that accurately classifies questions can significantly impact the quality of interview training by providing effective feedback. Distinguishing between recommended and not recommended questions is essential for delivering high-quality feedback.

Achieving a balanced accuracy of 87% in classifying these two categories is a notable accomplishment, surpassing the state-of-the-art results of 72% reported by Haginoya et al.^52^. Although the model has a weighted average F1 score of 0.87, there is room for improvement in its ability to predict closed questions correctly. Reducing the original 15 question types to two fundamental categories may have affected the results when converting the multi-class task to a binary classification task. Future work could focus on refining the model’s performance in classifying closed questions to further enhance the interview training experience. Furthermore, to accurately measure the learning impact of the system, a more extensive study is needed, where each participant conducts multiple interviews with a child. This approach would allow us to assess the quality of the interviews over time, similar to the pattern observed in the studies by Pomedda et al.^28,30^.

When the classification model was applied to the conversational data from the study, the results revealed that the interviews conducted by both CPS professionals and non-CPS participants were of satisfactory quality using a rather high percentage of open-ended questions. However, the results also indicate that the interviewers rely on a high frequency of closed-ended questions, which is not good according to best practice standards. Closed-ended questions should be minimized as children often respond with little information. In addition, these questions tend to be suggestive, prone to response biases, and problematic to be used in investigative interviews^58^. Nevertheless, after examining the data, it was observed that the quality of interviews conducted by CPS professionals was comparatively better than those conducted by non-CPS participants. The participants seemed to perform consistently across different settings, indicating that the interview environment may not have a significant impact on their overall performance. Further research with more participants is necessary to determine the most suitable environment for interview training, particularly in terms of QoE and learning effect.

Future research should prioritize improving the various quality aspects of our child avatar system to provide a more effective learning platform for interview trainees. Our objective is to develop a flexible and efficient interview training system that is conducive to effective learning. Our future work will mainly focus on enhancing the dialogue model to generate more realistic responses by leveraging the advances made in large language models (LLMs), in conjunction with the use of more realistic-looking avatars generated using neural radiance fields (NeRF) or generative adversarial networks (GANs). In addition, we will also focus on improving the quality of feedback and exploring how feedback can be effectively utilized to enhance the overall learning experience.

## Methods

### System architecture

**Figure 5:**
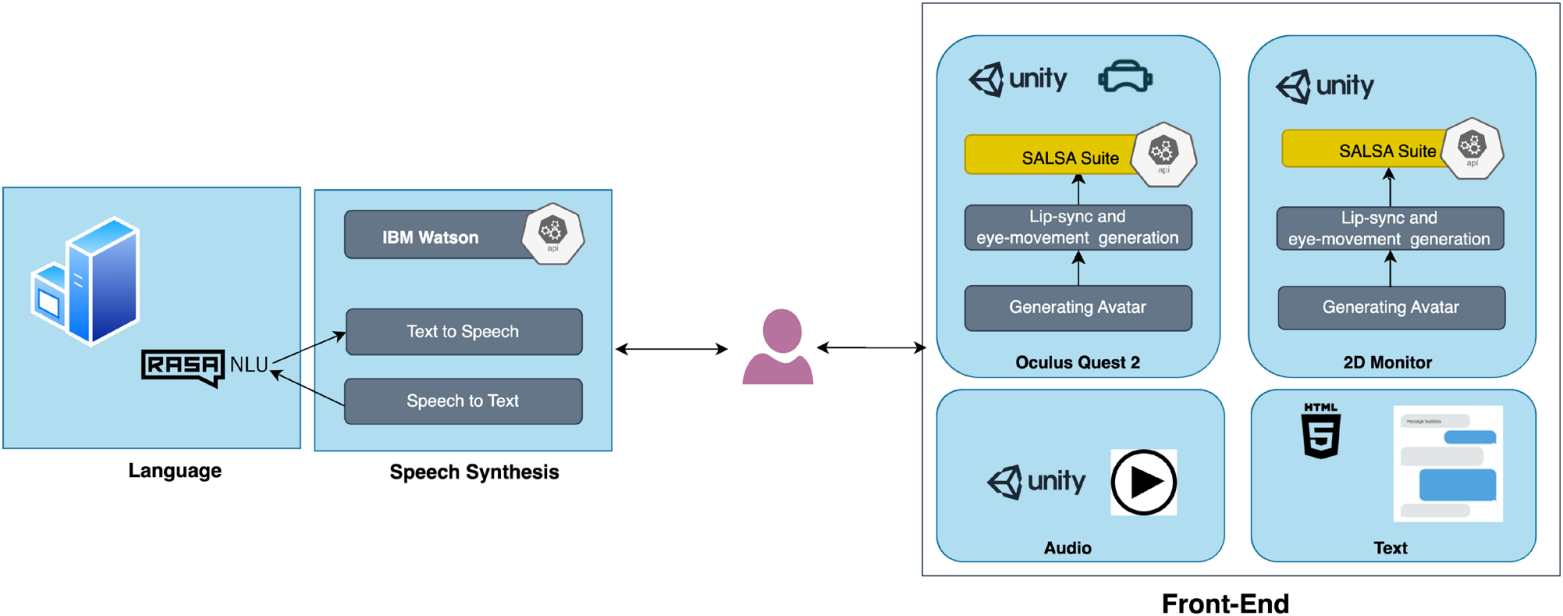
Architecture of the child avatar system for our comparative study with CPS professionals and students. (i) Language component incorporates a dialogue model that we developed using the RASA framework, which was trained on mock interviews, and a classification model for evaluating the quality of the interviews. (ii) Speech Synthesis component utilizes IBM Watson services for speech-to-text and text-to-speech synthesis. (iii) Front-end of the system provides four interactive environments: a VR environment created using Unity and Oculus Quest2, a 2D environment with audio also developed using Unity, and a text-based environment created using the RasaX platform.

In this section, we discuss the overall architecture of the interactive child avatar system architecture for conducting a comparative study with CPS professionals and students shown in Figure 5. The system consists of three main components: (i) The language component, which comprises a dialogue model that we developed using the RASA^59^ framework and a classification model that enables us to evaluate the quality of interviews; (ii) The speech synthesis component, which utilises IBM Watson services to provide speech-to-text (STT) and text-to-speech (TTS) synthesis^60^; and (iii) The front-end of the system, which offers four different interactive environments: a VR environment created using Unity and Oculus Quest2, a 2D environment and audio also developed on Unity. The text-based environment was created using the RasaX platform.

#### Language

The language component of the model is designed to analyze a child’s conversation and produce realistic and suitable responses to an interviewer’s questions. Furthermore, there is a classification model that can offer valuable feedback on the quality of interviews and assist in enhancing interview skills.

#### Dataset

The dataset consists of transcripts from interviews conducted by professionals according to specific guidelines, as part of a training program at the Centre for Investigative Interviewing, which is situated in Griffith University, Australia^61^. The transcripts consist of simulated dialogues between professional actors portraying 5-7-year-old children and an interviewer who could be from CPS or law enforcement. These conversations aim to replicate an investigative interview with a child who may have been a victim of abuse.

#### Dialogue model

The current dialogue model is specifically designed to apply the dialogical communication method (DCM)^62^, which is an investigative interviewing methodology currently employed in Norway. The Norwegian model is based on the research-based protocol developed by Michael Lamb and his colleagues at the National Institute of Child Health and Human Development (NICHD)^13^. The dialogue model was developed using an open-source framework of RASA which runs on tensorflow back-end. It has a modular structure with deep learning models, or rule-based techniques, which are employed to set up a complete dialogue model. Each module accepts the data in a predefined format, which requires quite a lot of pre-processing and data augmentation work for training. The Natural-language Understanding (NLU) module uses the Dual Intent and Entity Transformer (DIET)^63^ architecture to model a joint task of intent and entity recognition. It processes the input text and predicts the best possible intent from the training data along with entities in that input. The rule module is used to model fallback strategies or control the behaviour of the dialogue model where necessary. According to the rules defined, a specific appropriate action is performed if a certain intent is recognized. These actions are defined in the action modules. The story module is used to control the evolution of the conversation and control the flow of conversation based on questions asked based on slot values defined in the NLU module or some specific intent. If a user asks a leading question, the child avatar answers with a vague or unproductive reply with a certain probability. This is designed to reinforce the need of asking open-ended questions and communicate negative feedback to the user.

Table 7 presents an excerpt of a participant’s conversation during the study, with the participant’s name removed for privacy purposes. The conversation shows that interviewer started with a bit of introduction and rapport building and then transition into substantive phase. Table 7 don’t have the complete substantive as later child start talking about the alleged abuse.

**Table 7:** Example excerpt during the interaction between the user and a virtual child.

Conversation Excerpt	
User	Hi Hillary
Bot	Hello.
User	What do you want to talk about today
Bot	About Janet’s house.
User	Okay tell me about it
Bot	Janet’s house is big and she has a bubble pool in the garden. I go there for play dates, but I don’t want to go anymore.
User	Okay why don’t you wanna go anymore.
Bot	Because I don’t like her Pop.
User	Okay tell me why you don’t like her pop.
Bot	He’s Janet’s grandpa, but I call him Pop.
User	Okay you call him pop. Tell me about pop.
Bot	He has grey hair and he lives with Janet in their house and he’s a bit fat.
User	Okay he’s a bit fat and what does he do that you don’t like.
Bot	I just don’t like him because he did a rude thing.
User	A rude thing what did you do?
Bot	I went to Janet’s house, and it was really hot, so we were playing outside in the garden with the sprinklers and running through them and that was really fun.
User	...
Bot	...

#### Front-end

**Figure 6:**
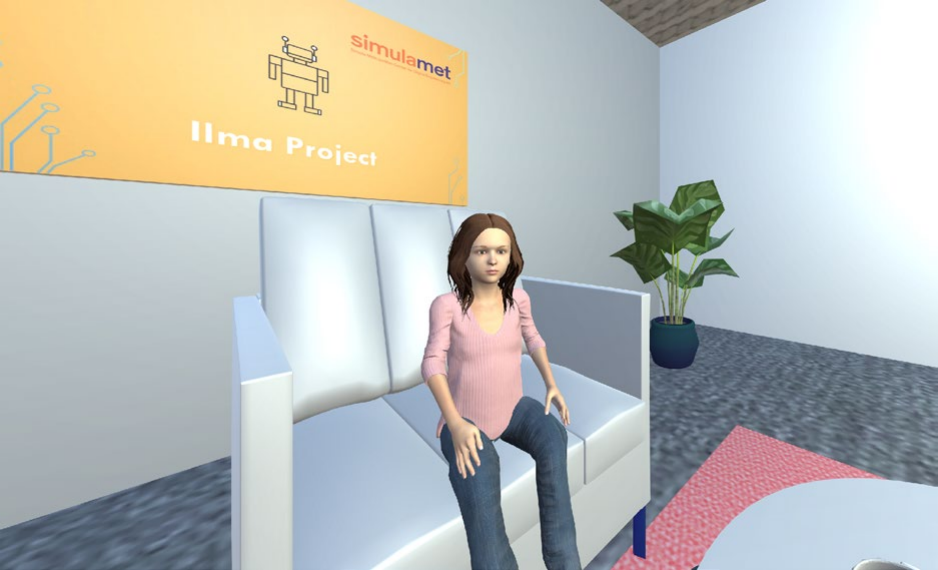
A snap-shot of the child avatar seen through the VR headset.

We used the system developed for our pilot study as the baseline^32^. The VR environment is running through an Oculus Quest 2 as a front-end. Three more interactive environments have been added to the current VR-based system. Thus, users can interact with the child’s avatar in four different environments. Regardless of the environments (front-end), the system’s back-end has the same dialogue model, which means that the responses generated by the virtual child remain consistent across different environments. The front-end of the child avatar system was developed in the Unity game engine in all environments except the text-only environment. RASA-X is used to develop a web interface for the text-only environment, where users have a textual conversation with the child. The 2D environment runs on a PC displayed using a 24-inch desktop monitor, and the audio-only environment was the same as the 2D with the difference that no visual elements were involved. The same child avatar character is used in both 2D and VR environments developed using the open-source project Unity Multipurpose Avatar (UMA)^64^ which allows customization for combining meshes and textures of the characters. The unity asset Salsa Suit^65^ was used to match the generated voices to the meshes and the texture of the avatar, to generate eye, head, and lip movements synced to the voices. In addition to facial movements created dynamically from the generated voice, a few pre-recorded animations were created by moving the meshes and the joints to create more natural movements and gestures in the avatar’s hand and neck^66^. The snap-shot from a VR interface of the child avatar is shown in Figure 6.

#### Speech synthesis

We use the IBM Watson services for STT and TTS synthesis. Watson STT and TTS cloud service APIs play the role of a communication bridge between the dialogue model (back-end) and interactive environment (front-end) components. In our pilot study, participants gave us feedback that audio did not match the child’s age^32^.IBM TTS services do not offer a child character option. However, users can utilize speech synthesis markup language (SSML) attributes and elements to manage text synthesis. To determine the most appropriate voice for a six-year-old girl, we conducted a small user study in collaboration with our colleagues in the social sciences, who are among authors of this paper. Nineteen distinct voices were developed, each featuring different female characters and prosody, which regulates pitch and speaking speed. The outcome of this study was the identification of the ideal audio representation of a six-year-old girl. The chosen voice’s character, prosody rate, and prosody pitch were established as Allison, slow, and x-high, respectively. Except for the textual environment, the user interacts with the front-end system verbally. Questions asked by the users are synthesised by the IBM STT API and then processed by the dialogue model to generate appropriate responses. These responses are then synthesised by the IBM TTS API, which is played to the user through either speakers or VR headsets. Although multiple characters are available in IBM TTS API, all of them are adults. We adjusted the speed and pitch of one of the female characters to generate a child-like sound, as the pilot study showed that an adult voice over a child character is not taken well by the participants.

### Classification model

To train the model, 58 transcripts were randomly selected and coded by a team of two psychologists and two research assistants. One of the authors, experienced with previous coding trained the other coders with best-practice guidelines and coding categories. Data was annotated with 14 different type of questions of open-ended (initial invitation, breadth prompt, depth prompt, descriptive, minimal encourager) and closed-ended (specific Yes-No, specific forced choice/option posing, leading, repeated question, leading, pressure, referring, indication, and visual). The coding process was conducted using the SIM and corresponding coding manual^67^.The coders established inter-rater reliability on a separate set of transcripts until they reached on moderate agreement regarding the question types with Cohen’s Kappa >0.70.

Training transcripts contained 2,745 turns of conversation. We focus on two higher-level classes: open-ended and closed questions. This was motivated by the the fact that we wanted to keep it simple for first model we train to do this task and also be able to compare our model performance in similar setting with Haginoya et al.’s model^52^.

Table 8 shows the distribution of classes,the number of open-ended and closed questions,in the training and test sets used to train. In the training set, there were 1,191 closed questions and 1,554 open-ended questions. Using these annotations, we fine-tuned the GPT-3 davinci model to predict the question type in the conversations. To evaluate the performance of the classification model, we used an additional 40 transcripts, which were also annotated. In the test set, there were 400 closed questions and 2,015 open-ended questions.

When generating predictions from the fine-tuned GPT-3 model and evaluating a classification model that predicts the question types in conversations, we used a temperature of 0.0 and set the maximum tokens to one.

**Table 8:** Training and test set class distribution.

Subset	No of transcripts	No of turns	No of closed	No of closed
Training	58	2745	1191	1554
Test	40	2415	400	2015

### Experiment details

The study had two parts. The first part was designed according to the recommendations of ITU-T P.809^50^ for short interactive test stimuli, which provides guidelines for conducting interactive subjective studies and assessing the QoE of a gaming system. We chose 90 seconds interactions to keep the study brief and for ease of recruiting participants who have domain knowledge. There are no strict recommendations for the duration of an ideal stimuli, it suggests that 30 seconds may not be sufficient to measure flow and immersion. For this, 10-15 minutes might be reasonable. We argue that 90 seconds could be enough for the comparison of different stimuli in the same setup and interactions can be enough to capture user experience for different system modalities. In order to validate this argument, part two was designed to evaluate the impact of the duration of the interactive session on the quality assessment metrics. Participants were asked to have an interactive session with a child avatar in VR without any time constraints.

In the first part of the study, we asked users to have a 90 second interaction in four different environments: text, audio, 2D, and VR. The order of these settings was randomized using the Latin squared design to avoid any potential sequencing bias. The primary objective of this research was to gauge and compare the QoE across various settings while minimizing any impact the sequence of settings may have on user experience. We also did not measure the quality of the dialogue model or the impact of the dialogues on QoE of each environment, for that purpose response generation from the dialogue model was kept constant across all settings. The second part of our study entailed participants interacting with a child avatar in VR with no time limit. It is referred as *long VR* in the result section to avoid ambiguity.

#### Ethical approval

- We affirm that our data collection process complies with the General Data Protection Regulation (GDPR). We have maintained data accuracy in accordance with Article 5(1)(d), ensured data integrity and confidentiality in accordance with Article 5(1)(f), and implemented robust security measures as required by Article 32. We have adhered to purpose limitation (Article 5.1 b), collecting data only for explicit, legitimate purposes, and not processing it for incompatible uses. We have practiced data minimization (Article 5.1 c), processing only adequate, relevant, and necessary data. Finally, we’ve enforced storage limitation (Article 5.1 e), not retaining data longer than necessary for its intended purpose.
- This study has received approval from the Norwegian Agency for Shared Services in Education and Research (SIKT) and the Data Protection Officer, OsloMet. No personal data was stored. SIKT Application number is 614272.
- We affirm our ethical commitment to ensure legality, fairness, and transparency (Article 5.1 a) by providing clear information and obtaining consent for data processing. Before beginning, participants were informed about the nature of their conversation with the virtual child and the potential adverse effects of prolonged VR use, such as dizziness or cyber-sickness. They signed a consent form allowing their feedback to be stored for research purposes, with the option to withdraw consent at any time in the future. The entire study took approximately 40 minutes for participants to complete. Participants were given a 200 Norwegian Kroner gift card for their voluntary participation.

Study participants were recruited in April 2022. The recruitment was specifically designed to target two groups: students specializing in child welfare psychology at Oslo Metropolitan University (Oslomet), and professionals currently employed in Child Protective Services (CPS). Information about the study was disseminated to these demographics to ensure a comprehensive representation of individuals with relevant knowledge and experience in child welfare. The study was executed during the time span from April to May of 2022, specifically during weeks 17 through 19. All participants were invited to attend at the SimulaMet research facilities, where they participated in the study. The data collected from the user study underwent an initial processing phase in July 2022, contributing to our preliminary work^53^. Subsequently, an additional round of data processing was performed in December 2022 and January 2023, purposed to facilitate the current extension of our work.

#### Storyline

To choose a storyline for the experiment, the interview transcripts were categorized into different personas. The persona selected for this study was determined by the abundance of transcripts and the extent of information that could be used to generate dialogues of sufficient length. The chosen persona was named Hillary and had the following background:

*Hillary is a 6-year-old girl whose cognitive, emotional, social, and physical development appears to be typical for her age. She has several close friends at school, including one named Janet. Hillary lives with her biological parents and a younger brother. She has just started her first year of school and appears to have adapted well. Occasionally, she visits Janet at her home for play dates. Janet lives with her biological parents and her grandfather, whom the children refer to as “Pop.” If Janet’s parents are still at work, Pop usually takes care of the children. During these visits, Hillary and Janet usually play indoors and outdoors in the garden until Hillary’s parents come to pick her up and take her home. A few days ago, while Hillary was in the car with her mother after a visit with Janet, she told her mother that Pop did something inappropriate to her and touched her private parts while they were in the pool in the garden. As a result, Hillary has come to the interview to discuss this incident.*

#### Demographic of the participants

In this research, a total of 21 participants took part, consisting of 20 females and one male. Their ages ranged from 20 to 59 years, with a mean age of 31.2 years and a standard deviation of 9.7. To begin with, they were requested to provide their ratings on their familiarity with interviewing children and VR. Approximately 60% of the participants had one or more years of experience as a CPS professional. Nonetheless, despite their expertise in CPS, most of them (75%) had no prior experience with VR. Each participant interacted with a child avatar in all environments, and none of them reported experiencing any form of motion sickness while utilizing the prototype.

#### Questionnaire

In addition to a question measuring the overall user QoE, we included three questions regarding *responsiveness* with GIQS^68^, three questions regarding *realism* of the avatar inspired by the work conducted by Wilson et al.^69^, two questions for measuring *flow* from GEQ^70^, three questions for *presence* from IPQ^71^, and three questions measuring the learning effects, engagement in learning, and self-efficacy as follows.

- *Communication skills* using this system aids me in acquiring knowledge and skills in interviewing maltreatment children.
- *Engagement* using this system would increase my engagement in the learning process.
- *Self-efficacy* using this system can improve my self-efficacy.

The questionnaire was answered on a 7-point Likert scale. The post-questionnaire is shown in Table 9 to measure the post-experience and user preference as the environment.

**Table 9:** List of post-questionnaire items.

Post experience	
Q1: Which environment felt more realistic for you?	
Q2: Which environment do you like the most?	
Q3: Which environment do you prefer to use?	
Q4: Which environment creates the best experience for you?	

## Data Availability

All the subjective data collected during the user studies of this paper are available at https://docs.google.com/spreadsheets/d/e/2PACX-1vS8BzPnV7YbZxcQjcPjlN0EIYomA5E5qzmMzz3rNxUnqovoQiGEU0k1ZUPHwPsjSQ/pub?gid=2002569374 &single=true &output=csv. Although, the implementation details of the dialogue model on RASA and the UNITY 3D model of the visual avatar can be provided upon reasonable request. However, we regret to inform you that due to the ethical considerations and privacy concerns associated with the sensitive nature of the conversational data - specifically related to alleged sexual abuse - we cannot make the models and implementation of the child avatar trained on this data publicly available. Additionally, we would like to clarify that our classification model relies on a proprietary API key from OpenAI, which prevents us from sharing the model. Correspondence and requests for materials should be addressed to S.Z.H and S.S.S.
